# A Comparison of Spectral Angle Mapper and Artificial Neural Network Classifiers Combined with Landsat TM Imagery Analysis for Obtaining Burnt Area Mapping

**DOI:** 10.3390/s100301967

**Published:** 2010-03-11

**Authors:** George P. Petropoulos, Krishna Prasad Vadrevu, Gavriil Xanthopoulos, George Karantounias, Marko Scholze

**Affiliations:** 1 Department of Earth Sciences, University of Bristol, Queens Road, BS8 1RJ, Bristol, UK; E-Mail: marko.scholze@bristol.ac.uk; 2 InfoCosmos, Pindou 71, 13341, Athens, Greece, http://www.infocosmos.eu/; 3 Agroecosystem Management Program, Ohio Agricultural Research and Development Center, The Ohio State University, Wooster, OH 44691, USA; E-Mail: vadrevu.2@osu.edu; 4 National Agricultural Research Foundation, Institute of Mediterranean Forest Ecosystems and Forest Products Technology, Terma Alkmanos, Ilisia, 11528 Athens, Greece; E-Mail: gxnrtc@fria.gr; 5 Department of Natural Resources Development and Agricultural Engineering, Agricultural University of Athens, Iera Odos 75, 11855, Athens, Greece; E-Mail: gkarant@aua.gr

**Keywords:** Landsat TM, burnt area mapping, Artificial Neural Networks, Spectral Angle Mapper, Greek forest fires 2007

## Abstract

Satellite remote sensing, with its unique synoptic coverage capabilities, can provide accurate and immediately valuable information on fire analysis and post-fire assessment, including estimation of burnt areas. In this study the potential for burnt area mapping of the combined use of Artificial Neural Network (ANN) and Spectral Angle Mapper (SAM) classifiers with Landsat TM satellite imagery was evaluated in a Mediterranean setting. As a case study one of the most catastrophic forest fires, which occurred near the capital of Greece during the summer of 2007, was used. The accuracy of the two algorithms in delineating the burnt area from the Landsat TM imagery, acquired shortly after the fire suppression, was determined by the classification accuracy results of the produced thematic maps. In addition, the derived burnt area estimates from the two classifiers were compared with independent estimates available for the study region, obtained from the analysis of higher spatial resolution satellite data. In terms of the overall classification accuracy, ANN outperformed (overall accuracy 90.29%, Kappa coefficient 0.878) the SAM classifier (overall accuracy 83.82%, Kappa coefficient 0.795). Total burnt area estimates from the two classifiers were found also to be in close agreement with the other available estimates for the study region, with a mean absolute percentage difference of ∼1% for ANN and ∼6.5% for SAM. The study demonstrates the potential of the examined here algorithms in detecting burnt areas in a typical Mediterranean setting.

## 1. Introduction

Over the past few decades, wildland fire research has been receiving increasing attention in several regions of the world, including Mediterranean regions, because of the wide range of ecological, economic, social, and political impacts of such fires. Vegetation fires are spread throughout the different biomes, in both natural and managed ecosystems and may profoundly alter the structure of the landscape affecting ecological processes [1]. Being able to obtain accurate as well as rapid mapping of burnt areas is of key importance to both environmental scientists and policy makers. Such information is very important, for example, for estimating the economic consequences from the fire and establishing rehabilitation and restoration policies in the affected areas, thus assisting to avoid post-fire hazards and long-term degradation [2]. Accurate knowledge of the geographical and temporal distribution of the fires is also vital in modelling the atmospheric and climatic impacts of biomass burning and estimating the total emissions from it [3]. Burnt area delineation on an operational basis can also provide important information on land cover changes related to ecology and biodiversity, that can in turn significantly assist in understanding post-fire recovery of an affected area [4].

Satellite remote sensing is increasingly being used as a practical solution for the rapid and cost-effective evaluation of impacts from wildfires [5]. The general circumstances that make remote sensing attractive for this purpose include its ability to provide inexpensively and repetitively synoptic views of large areas, at a spatial resolution suitable for regional and global fire analysis studies, even on inaccessible locations. Burnt area mapping by remote sensing is mostly done through measuring the changes of the reflectance of surface objects after the fire that occur mainly in the reflective part (0.45–3.0 μm) of the electromagnetic spectrum. This is because a fire alters the spectral response of the land surface by reducing the fractional cover, density, greenness and water content of vegetation by partially or completely removing surface litter, exposing and altering simultaneously the colour and brightness of the soil [6]. These changes in the surface properties result to a strong contrast of the fire-affected areas with the surrounding environment [7,8].

Generally, remote sensing-based methods employed today differ with regards to the number of the satellite scenes used and the type of processing carried out in the analysis. Regarding the number of images employed in the analysis, methods are separated into single-image methods if one post-fire image is used, and bi-temporal methods when more than one image (usually one image before and one after the fire) is used. Single-image approaches have important advantages over bi-temporal methods, mainly because they are more cost and time effective in processing and analysis, and they are less prone to potential processing errors [9]. In addition, these methods do not require registration and do not require correcting the satellite imagery for errors arising from sun–sensor geometry, atmospheric effects or perhaps differences in sensor calibration [10]. Nonetheless, for this type of approach, satellite imagery has to be acquired very shortly after the suppression of the fire incident, as otherwise, the identification of the burnt areas becomes more difficult [11]. On the other hand, the main advantage of the multi-temporal approaches is that they reduce the confusion with the types of permanent cover [12]. In terms of the type of processing applied, methods employed vary from those based on the calculation of simple radiometric indices, such as the Normalised Difference Vegetation Index (NDVI) of [13] or the Normalised Burnt Ratio (NBR) of [14], to other more complex ones, such as image classification and sub-pixel analysis [15]. A few other studies have proposed the retrieval of burnt area from surface temperature derived from thermal bands of satellite sensors (e.g., [16]). Overviews on the different remote sensing-based approaches for burnt area mapping can be found in [17,18].

Based on the available methods and satellite sensors in orbit, operational services have also been developed by international space agencies providing at multiple resolutions and spatial scales fire analysis data, including burnt area maps, often in the form of regional-scale products [19]. Examples of such freely accessible operational services include the European Forest Fire Information System (EFFIS), the Moderate Resolution Imaging Spectroradiometer (MODIS) fire analysis products and the Risk-EOS emergency response service. EFFIS (http://effis.jrc.ec.europa.eu/) has been in operation by the Joint Research Centre (JRC) of the European Commission since 1997. It provides the EU countries with a daily map of fire risk and also burnt area maps of forest fires larger than 50 ha occurred in each Mediterranean EU country. Burnt area data from EFFIS are derived from the combination of the MODIS visible-near infrared (VNIR) and shortwave (SWIR) data (250 m and 500 m respectively) and the MODIS 1 km active fire product [20] as well as various ancillary data [21]. The MODIS burnt area product (MCD45A1—http://modis-fire.umd.edu/products.asp/) is one of the series of the MODIS fire analysis products, and includes monthly estimates of burnt area with a spatial resolution of 500 m. Burnt area estimates from MCD45A1 are based on the detection of rapid change in the MODIS daily reflectance values from Terra and Aqua platforms, assisted by a bidirectional reflectance model and by statistical assumptions of change probability from a previously observed state [22]. Risk-EOS service (http://www.risk-eos.com/actus/pge/index.php?arbo=0) was developed recently in the framework of the GMES-SE programme (Global Monitoring for Environmental Security/Service Element) of the European Space Agency. It is essentially a crisis response service to situations engendered by natural disasters, covering different stages of these from prevention to crisis management and damage assessment. Burnt area mapping by Risk-EOS involves the automatic production of highly accurate maps of burnt area, derived from implementation of various spectral indices combined with a change vector analysis. Production of these maps is generally based on the analysis of images obtained by more than one spaceborne system, depending on the detail and accuracy specified by user needs.

Depending on the combination of scientific approach and satellite sensor used, burnt area estimates can vary significantly. Various reasons can be attributed to these differences, including the influence of vegetation type, topography, soil conditions, geographical location as well as satellite sensor specifications and the potential limitations of the estimation methodology employed each time. As actual field-based burnt area measurements after the fire event can be cumbersome and costly to retrieve, validation of the satellite-derived burnt area maps has been based traditionally on the statistical analysis of classification accuracy assessment of the produced thematic maps (e.g., [23]). Thus, the necessity for the development and validation of methodologies able to produce accurate burnt area estimates from remotely sensed data on local, regional, and global scales constitutes an active research topic [2].

Landsat TM is among a wide variety of other sensors being used to estimate burnt area [24,25]. Landsat TM has a number of advantages that makes it unique for deriving burnt area estimates. This sensor is currently the only high spatial resolution sensor (30 m in the reflective channels and 120 m in one thermal channel that it has) providing, at no cost, global image data at high spectral resolution (7 bands from visible to thermal infrared), compared to other high spatial resolution radiometers of high acquisition cost (e.g., ASTER, ALOS, SPOT), or to freely distributed coarser spatial resolution imagery (e.g., MODIS, MERIS, AVHRR).

Landsat TM data have been used in the past in a large number of burnt area mapping studies at various geographical regions [18,26], some of which implemented in the Mediterranean region [17,27–29]. However, to our knowledge, the combined use of Artificial Neural Network (ANN) and Spectral Angle Mapper (SAM) spectral-based classifiers with Landsat TM imagery in burnt area mapping has been limited, if not existent, particularly so in Mediterranean conditions. In fact, ANN have been used in the past with Landsat TM data for deriving land use/land cover maps (e.g., [26,30] but not for obtaining burnt area cartography. SAM classifier have been combined with different multispectral and also hyperspectral sensors for deriving land use/land cover maps (e.g., [31–34]) and in burning assessment [8]; however, the use of SAM with Landsat TM in burnt area mapping has not been fully demonstrated as yet. The above is an important observation, particularly considering that these two classifiers have several advantages in comparison to other classifiers. More specifically, important advantages of ANN [35], include that: (1) they do not require assumptions on any probability distribution information on the data to be classified, (2) they are able to learn complex patterns which allows them to perform well particularly when the feature space is complex (or even incomplete) and the source data has different statistical distributions, (3) they can easily adapt to different types of data and input structures facilitating synergistic studies, (4) they are able to perform supervised classification using less training data than the maximum probability. Equally, SAM classifier, which is based on the computation of spectral angle similarity between a reference source and the target spectra, has a number of advantages over other commonly used spectral-based classifiers [36,37], namely: (1) it is not affected by solar illumination factors, because the angle between the two vectors is independent of the vectors length, (2) it is an easy and rapid method for mapping the spectral similarity of image spectra to reference spectra, (3) it is also a very powerful classification method because it represses the influence of shading effects to accentuate the target reflectance characteristics, (4) it does not require any assumptions on the statistical distributions of input data in performing classification (as is done in parametric methods, such as the Maximum Likelihood classifier). It should be noted however, that the relative insensitivity of the SAM classifier to brightness may be a reason why this classifier has not been so far used extensively for burnt area mapping. However, it is clearly worth investigating to which extent the reduced spectral mixing effects in the Landsat TM image may allow and the SAM classifier to perform well in burnt area mapping overcoming its brightness insensitivity disadvantage. Testing the SAM but also the ANN with Landsat TM data is of special interest because this sensor spatial resolution (30 m) decreases significantly potential errors introduced in the performance of this method from spectral mixing effects, allowing for deriving a “real” appreciation of the accuracy of these methods when used with satellite imagery, as the effect of mixed spectra is lower, compared to applying the methods with moderate resolution (250 m to 1 km) sensors such as MODIS.

The severe forest fires that occurred during the summer of 2007 in Greece [38] attracted a sustained interest by the remote sensing community in mapping the burnt area extent in the country, which is maintained until today [39–41]. The objective of the present study is to also build on these studies and assess for the first time the potential use of the ANN and SAM classifiers combined with cloud-free Landsat TM satellite imagery for obtaining burnt area cartography in a Mediterranean setting. The area of mount Parnitha, located close to the capital of Greece, is used as a case study site because in this region took place one of the most catastrophic fires during the summer of 2007. The ability of the two classifiers in delineating the total burnt area from the Landsat TM imagery, is evaluated based on the classification accuracy assessment of the derived thematic maps and on additional comparisons performed against the results, for the same fire, obtained by other agencies, specifically the World Wildlife Fund (WWF) Organization of Greece and the Risk-EOS operational service.

## 2. Study Site Description

**Figure 1.**
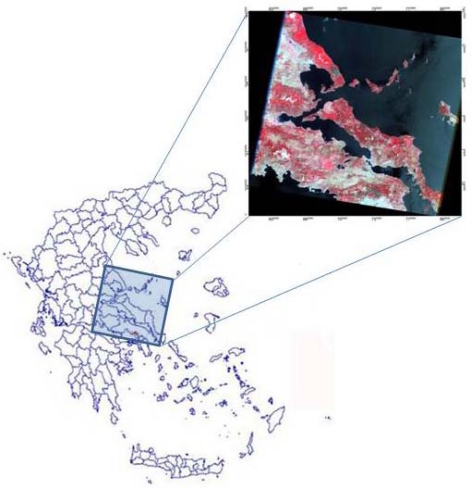
The location of the study site is shown on the left side of the map. The image on the right is the acquired false color composite Landsat TM scene (acquisition date: July 3^rd^, 2007).

The study area, Mt. Parnitha, is located approximately 30 km north of the city of Athens (Figure 1). Mount Parnitha is one of the four mountains surrounding the basin of Athens. Its tallest peak reaches 1,413 m. In 1961, Mt. Parnitha was proclaimed a National Park. Also, due to its biodiversity richness, it has been included in the European NATURA 2000 network of protected areas (http://www.natura.org/). At lower elevations it is covered mainly by Aleppo pine (*Pinus halepensis*) forest. At higher elevations, roughly above 900 m, the forest consists of an indigenous fir species (*Abies cephalonica*). The core of the National Park, at the top of the mountain covers an area of 38 km^2^, which is 90% covered by fir trees. The surrounding area of approximately 220 km^2^ is mainly covered by Aleppo pine. Below 300 m farmlands dominate to the north, whereas to the south and southeast the suburbs of Athens reach the base of the mountain.

On June 27^th^, 2007, at 19:30 local time, a fire, caused by sparks from an overloaded power line, erupted in the area of Dervenohoria, near a village called Stefani, approximately 15 km west of the core of mount Parnitha National Park. On the next day, fanned by a medium strength west wind, it entered the forested west slopes and canyons of the mountain and rushed to the top leaving only standing charred trees where it passed. Its main run stopped when it reached sparse vegetation on the east slope of the mountain in the morning of June 29^th^. Fought by strong forces with heavy ground and aerial fire fighting support, it was controlled three days later (July 1^st^, 2007).

## 3. Datasets and Methodology

Landsat TM imagery (path: 183, row:33) with an acquisitions date very shortly after fire extinction (July 3^rd^, 2007) was obtained from the USGS archive (http://glovis.usgs.gov/). This imagery was subsequently used for mapping the burning extent in the affected area (Figure 1, right). Selection of the imagery was based on the fulfillment of specific criteria, namely clear atmospheric conditions, high sun conditions, low water vapor, and temporal proximity to the fire event.

**Figure 2.**
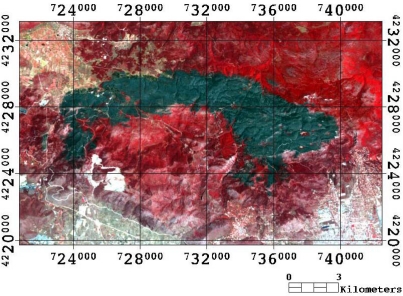
Subset of the Landsat TM post-fire imagery obtained on July 3^rd^, 2007 for the study region. The burn scar is shown in black.

The Landsat TM imagery was obtained at Level 1T, meaning that it had already been geometrically corrected and orthorectified. In deriving burnt area estimates from the obtained imagery, standard pre-processing procedures were subsequently applied. The Landsat TM imagery was imported into ENVI image processing software (v4.6) (ITT Visual Information Solutions SA) and the image georeferencing accuracy was initially checked with a reference map (1:10,000) that was available for the area. The positional accuracy was found to be within the level of the sensor pixel (RMS ∼ 30 m) of the map, which was considered satisfactory for the purpose of the present study. Then, to assist faster computational approach, a subset covering the region of Mt. Parnitha was extracted from the image using the ENVI subset function (Figure 2).

**Figure 3.**
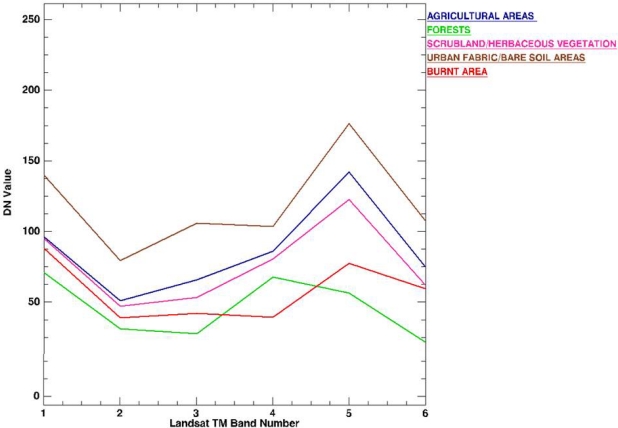
Average spectral signatures based on the selection of pixels selected for implementation of the SAM and ANN in the present study. DN is the pixels’ Digital Number recorded by the Landsat TM imagery which was used in the analysis.

In performing the image classification, the Landsat TM imagery was classified into five main classes: burnt area, agricultural areas, forests, scrubland/herbaceous vegetation, and urban fabric/bare soil areas. A stratified random sample of approximately 200 representative training points for each class were collected from the image subset for facilitating the implementation of the classifiers to the imagery. In addition, a separate training dataset of approximately 50 points for each class were collected for validating the derived classification maps. The same sets of sites were used in the implementation and validation of both the ANN and the SAM classifier. This allowed testing the effectiveness of both algorithms over the same post-fire Landsat TM scene. Selection of the training sites was based primarily on the CORINE 2000 100 m spatial resolution map for the study region, which was obtained at no cost (http://reports.eea.europa.eu/COR0-landcover/en). Training sites selection in the imagery was assisted by information derived from an available topographic map of the region and on screen inspection of the satellite imagery based on experience with the study region. The separability of the selected training sites for all classes was examined by computing their spectral separability in ENVI software. This function allows computing the spectral separability between selected regions of interest for a given input file, which is typically the satellite imagery from which the spectra have been collected. It is a measure of how well the selected and compared pairs are statistically separate. Computed values range from 0 to 2 where values greater than 1.8 indicate that the compared pairs have good separability, whereas very low values (less than 1) indicate that the compared spectra might be appropriate to be combined into a single one. In the present study, the six reflective bands of the Landsat TM post-fire image were used as the reference source for the computation of the separability index of the collected spectra from the training sites representing the different classes. Computation of the separability index was reported to be always higher than the value of 1.58 for all the class pairs compared. The mean spectra of each selected class are illustrated in Figure 3.

Following the selection of the training and validation sites, a supervised classification of the Landsat TM imagery was carried out. This was performed using the six reflective bands of the sensor, by applying the SAM and ANN classifiers to the Landsat TM scene as follows:

**A. Spectral Angle Mapper (SAM):** SAM is a spectral classifier that is able to determine the spectral similarity between image spectra and reference spectra by calculating the angle between the spectra, treating them as vectors in a space with dimensionality equal to the number of bands used each time [36,42]. Reference spectra for implementation of the technique can be taken either from laboratory or field measurements or can equally be extracted directly from the satellite imagery. In a n-dimensional multispectral space a pixel vector has both magnitude (length) and an angle measured with respect to the axes that defines the coordinate system of the space [43]. In SAM, only the angular information is used for identifying pixel spectra, as the method is based on the assumption that an observed reflectance spectrum is a vector in a multidimensional space, where the number of dimensions equals the number of spectral bands. Small angles between the two spectrums indicate high similarity and high angles indicate low similarity, whereas pixels with an angle larger than the tolerance level the specified maximum angle threshold are not classified [44]. The thresholding value is expressing essentially the maximum acceptable angle for the separation between the end-member spectrum vector and the pixel vector in the number of bands of dimensional space (again, here the six reflective bands of the Landsat TM image). Pixels with values higher than this threshold value are not classified. In the present study, SAM was implemented in the ENVI v4.6 image processing environment using a single value of 0.4 radians as the maximum thresholding value for all classes, after examination of different angle values (Table 1). The n-dimensional space was defined by the six reflective bands of the Landsat TM scene used in the analysis.

**Figure 4.**
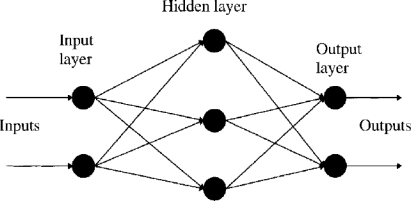
A representation of the general structure of an ANN, here with one hidden layer (adopted from [46]).

**B. Artificial neural networks (ANN):** ANN belongs to artificial intelligence techniques, which are widely used computing tools in image analysis. According to [45] an ANN is a massively parallel distributed processor made up of simple processing units, which has a natural propensity for storing experiential knowledge and making it available for use. A typical ANN comprises a large number of simple processing units, called nodes, linked by weighted connections according to a specified architecture. The basic ANN model consists of an input layer, a hidden layer and an output layer (Figure 4). Learning occurs by adjusting the weights in the node to minimize the difference between the output node activation and the output. One can select the number of hidden layers to be used and can choose between a logistic or hyperbolic activation function. For the implementation of ANN a number of parameters is required to be set. These include the training rate, the training threshold contribution, the training momentum, the training RMS exit criteria field and the number of hidden layers to be used. The training rate determines the magnitude of the adjustment of the weights. A higher rate will speed up the training, but will also increase the risk of oscillations or non-convergence of the training result. The training threshold contribution determines the size of the contribution of the internal weight with respect to the activation level of the node and it is used to adjust the changes to a node’s internal weight. The training algorithm interactively adjusts the weights between nodes and optionally the node thresholds to minimize the error between the output layer and the desired response. The training momentum is used to define the step of the training rate and its effect is to encourage weight changes along the current direction. Generally, a value greater than zero allows setting a higher training rate without oscillations. The training RMS exit criteria field defines the RMS error value at which the training should stop. The number of hidden layers defines whether the different input regions will be linearly separable with a single hyperplane or not. With no hidden layers (a value of 0), the different input regions are defined as being linearly separable with a single hyperplane, whereas if a higher value is used non-linear classifications are performed. The majority of the ANN architectures used in remote sensing are based upon a single hidden layer, but some authors have also used in land cover classification networks with two hidden layers (see review by [35]). Reference [35] also report that a single hidden layer should be sufficient for most problems, especially for classification tasks, because a multilayer perception with one layer can approximate any continuous function. Detailed descriptions of the definitions of the ANN parameters with reference to their concepts, architectures and learning algorithms, as well as their potentials and limitations are provided by [35,46,47].

In the present study, a multi-layered feed-forward ANN using a logistic activation function was implemented to delineate the total burnt area from the Landsat TM imagery in ENVI software. In the implementation of the ANN, a training threshold contribution value of 0.9, a training rate of 0.2, a training momentum of 0.9 and a training RMS exit criteria of 0.1 were used. The number of training iterations was set to 1,000 and one hidden layer was used. The classification statistics together with image segmentation on the classification images produced from the two classifiers, allowed delineating the burnt scar estimate by the two classifiers from the Landsat TM scene.

Classification accuracy for the burnt area map produced from each classifier was assessed using the error matrix, including the user/producer’s accuracy and omission/commission error, as well as the overall accuracy and the Kappa statistic [23]. The Kappa statistic provides a measure of the difference between the actual agreement between reference data and the classifier used to perform the classification *versus* the chance of agreement between the reference data and a random classifier [48]. Compared to the overall accuracy, the Kappa statistic has the advantage that it is computed using the non-diagonal values of the image classification, which means that it accounts for omission and commission in the classified data [48]. The producer’s accuracy indicates the probability that the classifier has correctly labeled an image pixel whereas the user’s accuracy expresses the probability that a pixel belongs to a given class and the classifier has labeled the pixel correctly into the same given class. Commission error (expressed as %) is derived by differentiating the user’s accuracy (also expressed as %) from 100, whereas the omission error (%) is computed by differentiating the producer’s accuracy (%) from 100 [49].

To determine the classification accuracy in the burnt area delineation the burnt area estimates derived from ANN and SAM were compared to independent satellite-derived burnt area estimates obtained from two different studies, which were available for the study region. The first was a study conducted by the World Wildlife Fund (WWF) of Greece and the other was the official burnt area estimate obtained from the Risk-EOS emergency response service. The burnt area estimate from WWF Greece had been derived from the analysis of orthorectified IKONOS panchromatic image (1 m spatial resolution, 4 pan-sharpened VNIR spectral channels) by applying a method based on NDVI difference. Their results, according to their report, had been checked against field surveys [50]. The Risk-EOS burnt area estimate had been obtained from the implementation of an NDVI threshold algorithm to SPOT (XS) multispectral imagery (20 m spatial resolution, 3 spectral bands in the VNIR) [40].

## 4. Results and Discussion

**Figure 5.**
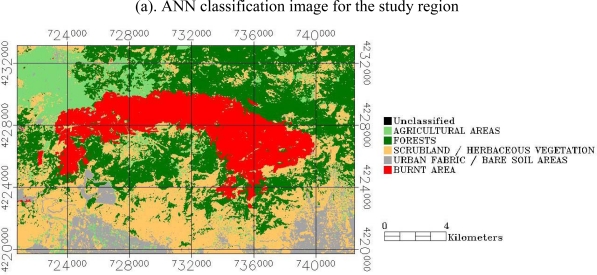

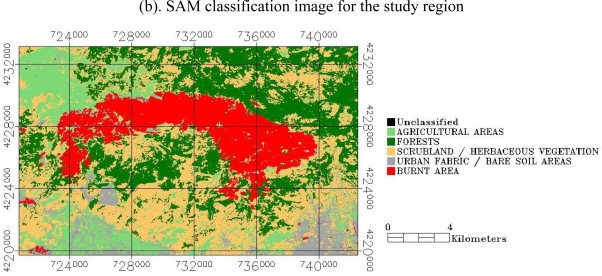
Classification maps obtained from Landsat TM post-fire imagery using ANN (a) and SAM (b) classifier.

The classification maps produced from the implementation of the ANN and SAM classifiers are illustrated in Figure 5 and the associated classification accuracy statistics are summarized in Table 2. For the burnt area class SAM returned a producer’s accuracy of 100%, suggesting that all of the collected validation samples were also found to belong in the same class. For the same class, user’s’ accuracy was reported as 98.72%, meaning that 98.72% of the points classified as burnt area can be expected to be burnt area when a field survey is performed. The classification of the burnt area class by the ANN showed higher producer’s and user’s accuracy than the SAM, with a producer’s and user’s accuracy of 100%. The producer’s and user’s accuracy of the classification of all classes was reported to be always higher than 65.85% and 60% for ANN and SAM, respectively.

For the SAM the classes with the highest producer’s accuracy were those of forests (100%) and agricultural areas (75%) followed by the urban fabric/bare soil areas (69.1%), whereas the lowest producer’s accuracy was obtained for the class of scrubland/herbaceous vegetation (65.85%). User’s accuracy was higher for the urban fabric/bare soil area (97.44%) and the forests (96.77%) followed by the agricultural areas (67.06%), the lowest user’s accuracy was found for the scrubland/herbaceous vegetation class (60%). For the ANN, highest user’s accuracy was found for the forests (100%) and the scrubland/herbaceous vegetation areas (92.68%), whereas lowest accuracy was reported for the urban fabric/bare soil areas (89.09%) and the agricultural areas (72.37%). User’s accuracy was higher for the forests (100%) and the urban fabric/bare soil areas (89.09%) followed by the agricultural areas (85.94%) and the scrubland/herbaceous vegetation class (71.70%). In general, both the forest and burnt area classes were clearly separable in both methods implemented herein. On the other hand, classes with relatively poor or ambiguous producer’s as well as user’s accuracy were for the case of the SAM classifier the scrubland/herbaceous vegetation and urban fabric/bare soil areas, whereas for the ANN the agricultural areas.

Overall, the ANN showed the highest overall classification accuracy (90.29%) and Kappa coefficient (0.878). The SAM classifier showed an overall classification accuracy of 83.82% and a Kappa coefficient of 0.795. The higher overall accuracy results from the ANN classifier, which are reported in Table 2, suggest that Landsat TM data when implemented with the ANN provide slightly better results in comparison to the SAM. In overall, classification results from the implementation of both the ANN and SAM reported here are generally also in agreement with results reported by other authors applying other classifiers (e.g., Maximum Likelihood with Landsat TM data for deriving land use land cover maps, in other regions. For example, [33] performed a comparison of different classifiers using airborne hyperspectral image data and reported classification accuracies of 84% and 50% for ANN and SAM respectively. [34] for a site in India evaluated the suitability of SAM and Maximum Likelihood algorithms with MODIS data for classifying multispectral data reported overall accuracies for the SAM method ranging between 70 to 76%. [26] implemented ANN with Landsat TM imagery for land use/land cover mapping using 6 classes for a region in California, USA, and reported an overall accuracy and Kappa coefficient ranging from 71 to 78% and 0.60 to 0.67% respectively.

**Figure 6.**
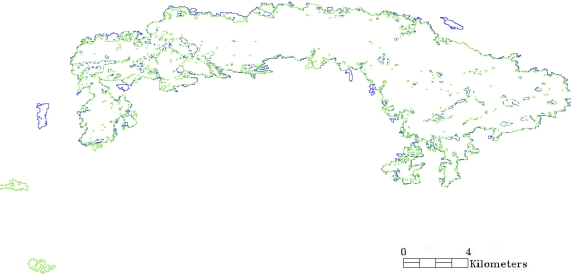
Differences in the burnt area estimation between the ANN (blue) and the SAM (green) classifiers.

Regarding the burnt area estimates derived from the implementation of the two classifiers to the Landsat TM imagery, SAM produced somehow a lower estimate (44.88 km^2^) than the ANN (47.78 km^2^). Figure 6 illustrates the overlaps and differences in the burnt area estimates between the two classifiers. From an operational perspective, the two techniques, as also seen from Figure 6, produced very similar results, apart from some obvious misclassifications of burnt area by the SAM in the south-west part of the studied region. It is also interesting to note that the differences between the two classifiers appear mainly in the areas of the borders of the burn scar, particularly the southern part of the detected scar (Figure 6).

Availability of total burnt area estimates from other sources for the study site allowed performing an intercomparison with the estimates of the present study (Table 3). Total area burnt by the ANN was clearly relatively closer to the estimates of burnt area obtained from earlier studies. The ANN-based burnt area estimate differs by 2.9% and 0.52% from the Risk-EOS and WWF Greece burnt area estimates respectively. The burnt area estimate from the SAM classifier differs by 8.8% and 5% from the Risk-EOS and WWF Greece burnt area estimates respectively. Since the same number of training samples for SAM as well as for ANN was used, our results clearly suggest that for the Landsat TM data, the ANN performs better than the SAM algorithm. This is certainly the case in our set-up. Thus, the results obtained from this study clearly demonstrate the ANN potential in delineating burnt areas in complex topography and ecosystem conditions, such as those that are often found in the Mediterranean region.

## 5. Conclusions

Observations from the Landsat TM sensor combined with either the SAM or the ANN classifier have been used in the past and at different geographical regions demonstrating the ability of these classifiers in land use/land cover mapping applications. The objective of the present study was to explore, for the first time, the potential use of these classifiers combined with the Landsat TM imagery analysis for deriving total burnt areas in a test site representative of a typical Mediterranean setting. As a study region the area of Mount Parnitha was selected, as this was one of the regions greatly affected by the catastrophic fires that occurred in Greece during the summer of 2007. Implementation of both the ANN and SAM was done using the same set of training and validation points selected over the acquired post-fire Landsat TM imagery, which allowed a direct comparison of their performance. Selection of training points was based principally on the CORINE 2000 (100 m spatial resolution) land nomenclature and ancillary information available for the study region. Evaluation of the ability of the two algorithms in delineating the total burnt area was based on the classification statistics of the produced thematic maps. In addition, the total burnt area delineated from each classifier was compared to earlier estimates for the site available from the Risk-EOS crisis response service and from a study conducted by WWF Greece, based on the analysis of SPOT XS and IKONOS imagery respectively.

Analysis of the classification statistics indicated that the ANN outperformed the SAM classifier in terms of the overall accuracy of the thematic maps produced by each classifier. Overall accuracy and Kappa coefficient was reported ∼84% and 0.795 for the SAM and 90% and 0.878 for the ANN classifier respectively. Burnt area estimates from both SAM and ANN were comparable to earlier estimates from WWF Greece and Risk-EOS. However, in comparison to SAM, total burnt area estimate from ANN was in closer agreement with the Risk-EOS (0.5% absolute difference) and the WWF Greece (1.42% absolute difference) burnt area estimate. For the latter, absolute difference was 8.8% and 5% from the WWF Greece and Risk-EOS estimates respectively.

The lower overall classification accuracy as well as the lower total burnt area estimate of SAM may be attributed to the relative insensitivity of this method to brightness variations, as the latter is not responsive to the effects by solar illumination, since this classifier uses only the angle between the two compared spectra and not the vector length. Indeed, as was also seen from the SAM implementation in this study, selection of the angle threshold value had an important effect in the overall classification accuracy as well as the estimated total burnt area. The choice of the angle value, along with the spectral range and the number of bands used were found to have an appreciable effect in the classifier performance. This is reasonable, as these parameters have a direct influence on the albedo value of the spectra that is to be classified with respect to the reference spectra. Also, SAM implementation is generally based on the assumption of end-members representing pure spectra of each class. Spectral confusion in pixels, if existing, may in turn potentially lead to classification errors for a spectral class by this method. This problem generally increases as the spatial resolution of the sensor decreases, from high (e.g., IKONOS) to moderate resolution spatial data (e.g., MODIS), and as the heterogeneity and complexity of the surface to be classified increases, as for example often seen in Mediterranean landscapes. Results obtained from the SAM indeed indicate that more than the direction of vector is needed in order to separate well the different land cover types, particularly those that are spectrally similar in nature. There is a high likelihood that angular information alone will provide good separation when the pixel spectra from the different classes are well distributed in feature space, particularly if a large number of spectral bands is used (as in the case perhaps of a hyperspectral imagery).

On the other hand, ANN have generally been reported to perform more accurately than other techniques such as statistical classifiers, particularly when the feature space is complex (e.g., areas of complex topography, which concerns most of Mediterranean conditions) and the training data have different statistical distributions and in cases where classes are less separable (such as in cases when decision boundaries lies on the edge of the class distributions between two or more classes). Also, ANN in contrast to SAM, are based on a ‘learning from examples’ framework, making it possible to use them in situations where exact cause-and-effect relationships are not known. Studies comparing various classification algorithms have also shown that ANN are able to deal better with heterogeneity inherent in the land cover categories, as well as with the multi-modal nature of Landsat TM data [51]. Nevertheless, implementation of ANN requires a training phase, which can be computationally very expensive and which also might not always converge to a unique solution. Also, the overall performance of this classifier is very much dependent on their ANN architectural design (e.g., [35,52]). Furthermore, the complexity of ANN requiring many parameters to be defined could still be sub-optimal for fragmented landscapes, such as those found in the Mediterranean conditions.

Clearly, although the present study demonstrates the potential of the examined here classifiers to be used in burnt area mapping, further studies should be conducted in dissimilar settings representative of the full range of Mediterranean site characteristics examining the applicability of these methods for this type of application. Recent advances in remote sensing instruments, have resulted in the development of hyperspectral satellite sensors (e.g., EO-1 Hyperion), which allow the retrieval of spectral information in a large number of narrow and contiguous spectral bands. Such sensors, have created new challenges in retrieving land cover information, including burnt areas [53], and deserve attention for future investigations of this type.

## Figures and Tables

**Table 1.** Results obtained during experimentation of different spectral angle maximum threshold values for SAM classifier implementation.

**Classification method**	**Overall accuracy (%)**	**Kappa**	**Burnt area estimate (km^2^)**

1. SAM01 (angle = 0.1)	74.43	0.684	39.39
2. SAM02 (angle = 0.2)	82.52	0.779	45.06
3. SAM03 (angle = 0.3)	83.82	0.795	45.31
4. SAM04 (angle = 0.4)	83.82	0.795	45.32
5. SAM05 (angle = 0.5)	83.82	0.795	45.33

**Table 2.** Summarized classification results from the implementation of the SAM and ANN classifiers to the Landsat TM post-fire imagery.

	**ANN**		**SAM**	
**Land cover classes**	**Producer’s accuracy (%)**	**User’s accuracy (%)**	**Producer’s accuracy (%)**	**User’s accuracy (%)**
Agricultural Areas	72.37	85.94	75.00	67.06
Forests	100.00	100.00	100.00	96.77
Scrubland / Herbaceous Vegetation	92.68	71.70	65.85	60.00
Urban Fabric / Bare Soil Areas	89.09	89.09	69.09	97.44
Burnt Area	100.00	100.00	100.00	98.72
**Overall accuracy**	*90.29*		*83.82*	
**Kappa coefficient**	*0.878*		*0.795*	
**BURNT AREA (km^2^)**	*47.78*		*44.88*	

**Table 3.** Burnt area estimates comparisons between those derived in the present study and those from other studies that were available for the region.

**Burnt area estimates from earlier studies**	**Area burnt (km^2^)**	**Difference (km^2^)**		**Difference (%)**	
		ANN	SAM	ANN	SAM
Study of WWF Greece *(based on IKONOS)*	49.20	1.42	4.32	2.9	8.8
Risk-EOS *(based on SPOT4 XS)*	47.26	−0.52	2.38	1.1	5.0
